# Urethral substitution with ileum in traumatic bladder neck-vagina fistula

**DOI:** 10.4103/0971-9261.55159

**Published:** 2009

**Authors:** Lavanya Kannaiyan, Sudipta Sen

**Affiliations:** Department of Paediatric Surgery, Christian Medical College and Hospital, Vellore, Tamil Nadu-632 004, India

**Keywords:** Ileum, rectus muscle flap, urethrovaginal fistula, urethral reconstruction

## Abstract

A five-year-old girl presented with post traumatic urinary incontinence secondary to rupture of the bladder neck into the vagina. Operative repair included a midline exposure with resection of the symphysis pubis, separation of the bladder neck from the vagina, repair of the torn bladder neck and urethral substitution with ileum. Normal continence and voiding was achieved.

## INTRODUCTION

Pelvic trauma in the female child can result in total loss of the urethra and rupture of the bladder neck into the vagina.[1,2] We report a case where this was reconstructed with bladder neck repair and urethral substitution with a Monti tube, resulting in complete cure of incontinence without the need for intermittent catheterization.

## CASE REPORT

**Figure 1:**
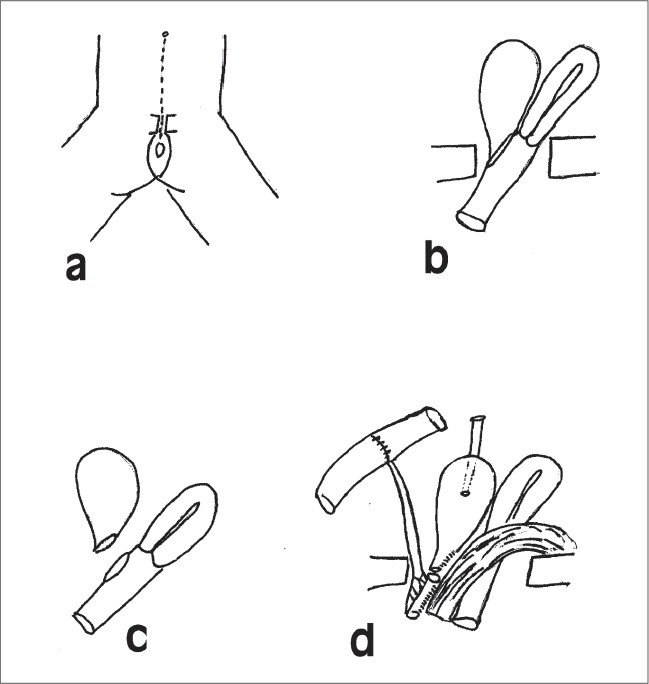
Schematic Diagram of the operative procedure. (a) Lower midline incision including the mons pubis and the anterior part of the vulval outlet. Anterior pubic symphysis excised. (b) Pathological anatomy exposed showing posterior rupture of bladder neck into the anterior vagina. Distal urethra destroyed by injury. (c) Surgical separation of bladder neck from the vagina. (d) Bladder neck repaired posteriorly and connected to Monti tube neourethra. Left lower rectus muscle flap placed between reconstruction and vagina to prevent refistulization. Appendicular Mitrofanoff also created.

A five-year-old girl presented with urinary incontinence following a history of pelvic trauma. She had a single orifice at the introitus from which she was continuously leaking urine. On endoscopic examination the bladder neck was found opening into the vagina. Operative reconstruction proceeded [Figure 1]. Operative exposure was obtained with a midline vertical incision from the umbilicus to the vaginal orifice with division and partial excision of the symphysis pubis. The remnant of the proximal urethra and bladder neck were dissected off the anterior vaginal wall at the area of the bladder neck-vaginal fistula. The rupture in the posterior aspect of the bladder neck was then repaired anatomically resulting in a normal appearing bladder neck with a short proximal urethral stump which would not reach the perineum.

A three cm segment of ileum was isolated on its pedicle to reach the perineum. The ileal segment was tubularized over an 8Fr feeding tube in the form of the Yang- Monti tube. It was then anastomosed proximally to the urethral stump and distally to the introitus at the site of expected urethral orifice. The lower half of the left rectus muscle was mobilized as a flap with its inferior attachment to the pubis left intact and carefully preserving the inferior epigastric vessels. This muscle flap was placed between the posterior bladder wall and the vagina to prevent refistulization.[3] The anterior vaginal wall was left unrepaired as the posterior and lateral vaginal walls were intact and closure of the anterior vaginal wall may result in stenosis. The anterior vaginal wall was left to reepithelialize over the rectus muscle interposed between the posterior bladder wall and vagina. An appendicular Mitrofanoff was also created opening at the umbilicus. We contemplate closing the Mitrofanoff port when the child desires it.

**Figure 2:**
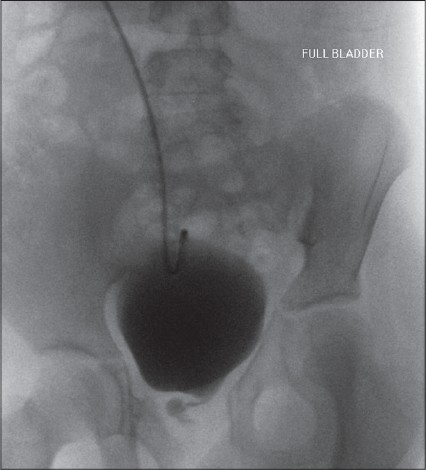
Post operative micturating cystourethrogram. Bladder was filled via the Mitrofanoff port and the patient voided via the reconstructed bladder neck and ileal neourethra.

At follow-up after 10 months, she is voiding normally with continent intervals of four to six hours. Residual urine measured after voiding was two ml. Ultrasound and serum creatinine remain normal and the micturating cystourethrogram was satisfactory [Figure 2]. She uses the Mitrofanoff channel once a day to maintain its patency.

## DISCUSSION

Traumatic posterior urethral loss in boys can be made up by mobilizing and rerouting the remaining urethra. The short female urethra is often totally destroyed in pelvic injury and has to be substituted.[1] Various methods have been described including – bladder and buccal mucosa, anterior bladder tube, ureters, appendix, anterior lateral thigh free flap, pedicled island skin, amnion grafts, fallopian tube, and colonic mucosa.[4–10] Tapered ileum has been used as a perineal Mitrofanoff stoma for patients with urinary incontinence of congenital origin.[11]

We have successfully used the Monti tube for posterior urethral substitution in boys.[12] It is more difficult to bring the appendix to the perineum based on a single blood vessel. It was easier to bring the appendix to abdominal wall. The Monti-Yang tube is more versatile in its blood supply and has further reach than the appendix. In the girl reported herein, we have used a similar method of urethra substitution after separation of the bladder neck from the vagina and repairing the posterior tear in the bladder neck resulting in complete continence without the need for intermittent catheterization. The continence of the bladder neck is due the anatomical repair of the injured bladder in a previously normal bladder, hence, resulting in a normally functioning bladder neck after the repair with a loss of urethra which was substituted. We have also used a rectus muscle flap to prevent vesicovaginal refistulization.
